# Interleukin-1-Beta and Dyslipidemic Syndrome as Major Risk Factors for Thrombotic Complications in Type 2 Diabetes Mellitus

**DOI:** 10.1155/2013/169420

**Published:** 2013-01-21

**Authors:** Oana Bădulescu, Codruţa Bădescu, Manuela Ciocoiu, Magda Bădescu

**Affiliations:** ^1^Department of Pathophysiology, University of Medicine and Pharmacy “Grigore T. Popa,” Universităţii Street, No. 16, 700115 Iaşi, Romania; ^2^Department of Internal Medicine, University of Medicine and Pharmacy “Grigore T. Popa,” Universităţii Street, No. 16, 700115 Iaşi, Romania

## Abstract

Diabetes mellitus (DM) is a complex disease characterized by chronic hyperglycemia, a known risk factor for accelerated atherosclerosis and vascular disease. The aim of this study was to show that the connection between DM and other risk factors, such as dyslipidemia, inflammatory phenomena, or the development of certain vascular injuries, leads to a high frequency of thrombotic events in diabetic patients compared to the nondiabetic population. The study included one hundred eighty patients divided in the following groups: diabetic without ischemic cardiopathy-related disorders (DM), diabetic with clinical or off-clinical (electrocardiogram, cardiac ultrasound) ischemic cardiopathy-related disorders (DM + IC), and nondiabetic with ischemic cardiopathy-related disorders (IC). We investigated the following parameters: von Willebrand Factor, HDL-cholesterol, LDL-cholesterol, interleukin-1-beta, protein C, and plasminogen activator inhibitor type 1. The results achieved in our study have revealed the highest thrombotic risk among the groups of diabetic patients, which is in direct correlation with the high values of interleukin-1-beta and the modifications of lipid parameters, acknowledging the data in the literature, according to which hyperglycemia alters endothelial functions directly and indirectly by synthesis of growth factors and cytokines and generates metabolic disorders which would explain the high risk for thrombotic events.

## 1. Introduction 

Diabetes mellitus, a disorder with a thrombophilic potential, became an endemic pathology worldwide, alarming not only because of its high incidence, but also because of its serious complications that lead to very costly medical assistance and even death. Numerous clinical observations [1] emphasized the frequent association of high blood pressure, DM, hyperlipidemia, and thrombotic complications, association that is likely to have a common pathogenesis-inflammatory endotheliopathy. 

Hyperglycemia may cause vessel damage through the following pathways: advanced glycation end product (AGE) formation, activation of protein kinase C (PKC), and sorbitol accumulation by way of the polyol pathway [2]. These pathways could be linked by an increased production of superoxide anions [3]. The hyperglycemia induced by increased oxidative stress and receptor for advanced glycation end products (RAGE) activation increases the activation of transcription factor-jB (NF-jB) in endothelial and vascular smooth muscle cells. This transcription factor regulates the expression of the genes encoding a number of mediators of atherogenesis such as leukocyte-cell adhesion molecules and chemoattractant proteins that recruit lymphocytes and monocytes into the vascular wall. Activation of the NF-jB pathway may also cause a switch of the endothelial functions toward a prothrombotic condition that, together with an altered platelet metabolism and intraplatelet signaling pathways and with the inflammatory sindrome, contributes to the pathogenesis of atherothrombotic complications in diabetes mellitus.

The physiopathology of the changes in the lipid metabolism of DM is multifactorial and incompletely deciphered. Quantitative anomalies of lipoproteins (LP) consist of increasing levels of triglycerides as the VLDL and IDL and decreased levels of HDLc due to the decrease of the subfraction HDL_2_. The decrease of HDLc is due to the rise in its catabolization. Qualitative anomalies of LP include changes in the size of LP (big VLDL particles, small and dense LDLc particles), increase of the contents of triglycerides in LDLc and HDLc, the glycation of apolipoproteins and lipids, and the increase of LDLc susceptibility to oxidation. These anomalies of LP alter their normal metabolism, increase their atherogenic capacity, and contribute to the promotion of accelerated atherogenesis in diabetic patients [4]. Small and dense LDLc fractions are the most atherogenous, increasing the risk of coronary disease up to three times.

The aim of this study was to show that the connection between DM and other risk factors, such as dyslipidemia, inflammatory phenomena, or the development of certain vascular injuries, leads to a high frequency of thrombotic events in diabetic patients compared to the nondiabetic population.

## 2. Materials and Methods

### 2.1. Patients

The study comprises 180 patients, 98 females and 82 males (Table 1), with ages between 61 and 82. The patients were followed between June 1, 2009 and December 1, 2009.

The patients were distributed in 6 groups (Table 2).

 Group 1: diabetic women with clinical or off-clinical disorders (electrocardiogram, cardiac ultrasound) related to ischemic cardiopathy (41 patients) (DM + IC).
 Group 2: diabetic women without ischemic cardiopathy-related disorders (37 patients) (DM).
 Group 3: nondiabetic women with ischemic cardiopathy-related disorders (20 patients) (IC).
 Group 4: diabetic men with clinical or off-clinical ischemic cardiopathy-related disorders (29 patients) (DM + IC).
 Group 5: diabetic men without ischemic cardiopathy-related disorders (33 patients) (DM).
 Group 6: nondiabetic men with ischemic cardiopathy-related disorders (20 patients) (IC).

The selection criteria of the diabetic patients were the presence of type 2 diabetes mellitus treated by diet and/or oral antidiabetic drugs and the duration of the disease between 2 and 10 years.

The patients with cardiovascular disorders have been included in the study based on the following criteria: the presence of signs and symptoms of any cardiovascular disease such as effort stable pectoral angina, aggravated pectoral angina, and chronic myocardial infarction; the values of systolic blood pressure >145 mmHg and of diastolic blood pressure >95 mmHg; the presence of arrhythmia of any kind: atrial and ventricular extra-systolic arrhythmias, supraventricular arrhythmias-atrial fibrillation, or atrial flutter of ischemic cause. In the study we also included the patients with the following cardiovascular disorders in personal pathological antecedents: coronary disease under any manifestation form, rheumatismal or non-rheumatismal valvulopathy, ischemic or hypertensive dilated cardiomyopathy, ischemic stroke.

### 2.2. Analyzed Parameters

von Willebrand Factor (vWF), protein C (PC), plasminogen activator inhibitor type 1 (PAI-1), and interleukin-1-beta (IL-1*β*) were determined using plasma; venous blood, collected in 0,105 M sodium citrate vacutainer (sodium citrate/blood ratio = 1/9), was centrifuged 15 minutes at 2500 rpm followed immediately by plasma separation and its freezing. Plasma obtained by centrifugation was frozen (no longer than 4 weeks) at −20 degree Celsius until the tests were performed.

- *von Willebrand Factor* measurement was performed by an immunoturbidimetry assay (vWF : Ag) on an automatic analyzer, using reagents from the Biomnis Laboratory, France. Reference values = 50–160%.
- *Protein C* was determined by an enzymatic immunoassay test (EIA) using reagents from the Biomnis Laboratory, France. Reference values: 65–140%.
- *PAI-1* was determined using a chromogenic substrate method with reagents from the Biomnis Laboratory, France. Reference values: <10 kU/L.
- *IL-1*β**: the test was performed using ELISA method. Reference values: <3.9 ng/mL.
- *HDL Cholesterol (HDLc)*: the venous blood collected in a vacutainer without anticoagulant, was centrifuged to obtain the serum, and the test was performed using an enzymatic colorimetric method on a TECAN microplate reader by commercially available kits (Audit Diagnostics, Ireland). Reference values: 65–100 mg/dL.
- *LDL Cholesterol (LDLc)*: was determined according to the Friedewald formula. Reference values: 0–130 mg/dL.

### 2.3. Statistical Data Interpretation

Data were expressed as mean $\left(\overset{-}{x}\right)$ ± standard deviation (SD).

*The Pearson correlation *(*r*) indicates the degree of linear dependence between the variables (and it ranges from −1 to +1). As it approaches zero there is less of a relationship (closer to uncorrelated). The closer the coefficient is to either −1 or 1, the stronger the correlation between the variables.

Unpaired Student's *t*-test was performed to determine whether there were significant (*P* < 0.05) differences between groups. The threshold of statistical significance which is unanimously accepted is 95%, namely, *P* = 0.05. The smaller the *P* value is than this value, the stronger the statistical significance (*P* > 0.05) statistically insignificant.

## 3. Results and Discussions

 The mean values of monitored parameters (Tables 3, 4, 5, and 6) have not shown significant differences between genders (*P* > 0.05) in any of the investigated groups.

### 3.1. Groups DM + IC

Within these groups of patients, the following parameters showed the most modified values: vWF, PAI-1, PC, and IL-1*β*. Regarding the distribution by gender, in females the values of the analyzed parameters have been slightly modified compared to those registered in males, without revealing a significant statistical difference between men and women.

The following significant correlations have been identified among the analyzed parameters.

- Females:
  - indirect correlations between:
    - vWF and PC (*r* = −  0.70),
    - HDLc and IL-1*β*  (*r* = −  0.70),
    - HDLc and PAI-1 (*r* = −0.53).
  - direct correlations between:
    - IL-1*β* and PAI-1 (*r* = 0.66).
- Males:
  - indirect correlations between:
    - LDLc and PC (*r* = −0.88).
  - direct correlations between:
    - IL-1*β* and vWF (*r* = 0.38).

According to the values we recorded, the plasma levels of vWF are considerably higher especially in the group of diabetic patients (DM + IC, DM − IC). The higher plasma levels are probably an expression of diabetes mellitus-related endotheliopathy. 

Regarding the von Willebrand Factor, currently no data exists supporting the assumption that the high plasma levels of the von Willebrand Factor represent a high risk factor for cardiovascular disease. However, it has been proved that in patients with angina pectoris this factor is predictive of later cardiovascular events, and it is also present in high levels in acute coronary syndromes [5]. Other researchers found high plasmatic levels of the vWF in different pathological states involving endothelial damage such as endothelial denudation and subendothelial structure exposure (acute and chronic renal failure, hypertension, diabetic nephropathy, and vasculitis) [6]. Persistent high values of this factor were also found in patients with uncomplicated diabetes mellitus or with diabetic nephropathy [7, 8].

In our study, the presence of direct correlation between IL-1*β* and PAI-1 (*r* = 0.66) supports the idea according to which the prothrombotic characteristics of the endothelium are also expressed under the action of some inflammation mediators (IL-l, tumor factor necrosis-TNF, etc.); recent evidence show that there is a close connection between the inflammatory processes and thrombosis, even when the endothelium suffers no actual morphological injuries [9]. Insulin stimulates PAI-1 synthesis in the hepatic cells and in smaller proportion in the endothelial cells, whereas endotoxins and IL-1, which have a proinflammatory effect, stimulate PAI-1 production in the endothelial cells but has no effect on it in the hepatic cells. The expression of proinflammatory cytokines and other mediators, including adhesion molecules, suggests that the inflammatory process may contribute to the vascular disease in diabetes mellitus. According to recent evidence, TNF-*α* plasma concentration is connected to the insulin resistance phenomenon, and it may be decreased by diet and weight loss, whereas IL-6 and C reactive protein (CRP) are known as having constantly high levels in type 2 DM [10]. All these compounds may induce a phenotype alteration of endothelial cells and/or monocytes able to trigger tissue factor production increase, which is the main procoagulant agent identified in the atheromatous plaque, to the further alteration of the coagulation and fibrinolysis mechanisms.

The indirect correlation between HDLc and PAI-1 (*r* = −0.53) supports the idea that in obese dyslipidemic patients with hyperinsulinemia the increase of the PAI-1 level leads to higher thrombogenic risk. It is well known that endothelial cells regulate fibrinolysis, as they synthesize and release both plasminogen activators (tisular plasminogen activator and substances such as urokinase) and PAI-1. 

Any quantitative or qualitative deficiency of highly anticoagulant molecules may be suspected in case of subjects with high prothrombotic risk [11]. This was probably the reason why protein C and protein S have been studied in DM. Yet, most of the results that made public so far are contradictory [12–14]. Some studies conducted on these factors revealed lower levels, whereas others detected normal values.

The results of our study shows that the diabetic patients were deficient in these factors. The lowest values were recorded in the group of diabetic patients with associated ischemic cardiopathy (DM + IC) followed by the group of diabetic patients with no cardiovascular diseases (DM − IC). This difference may be accounted for by the glycosylation phenomenon undergone by these proteins. The values of protein C have been indirectly correlated with vWF (*r* = −0.70), highlighting the increased thrombotic risk in this category of patients. In addition to its anticoagulant role, the active PC also increases fibrinolytic activity, both by stimulating the tisular plasminogen activator release from the endothelial cells and by neutralizing the PAI-1 activity. The sum of these effects of PC turns the PC system into one of the most important thrombosis protection mechanisms.

### 3.2. Groups DM − IC

In these groups of patients, we have obtained results that are similar to those underlined in the lot of the diabetic patients with cardiovascular pathology: increased values for the vWF, IL-1*β*, and PAI-1 and low values for PC.

The following most important correlations are noticed between the analyzed parameters.

- Females:
  - indirect correlation between:
    - PC and vWF (*r* = −0.70).
- Males:
  - indirect correlation between:
    - PC and PAI-1 (*r* = −0.30).

In accordance with our study results, as far as the diabetic population is concerned, the cardiovascular disorder risk is higher for diabetic women than for diabetic men; unlike the general population, where males are more frequently affected by the cardiovascular disorders, the male gender being even construed as a risk factor. The dyslipidemic syndrome occurs as a risk factor for the macrovascular disease in the case of diabetic women, especially those belonging to the second age category, an aspect that may be connected to the disappearance of hormonal protection, the occurrence of obesity, and to the duration of the disease.

The mechanism linking elevated levels of von Willebrand Factor FVIII to insulin resistance and type 2 diabetes may be related to the presence of endothelial dysfunction and/or inflammation, both involved in the development of insulin resistance [15, 16]. Endothelial cell incubation with high concentrations of glucose led to the increase of the vWF contents. Therefore, the high level of vWF in diabetics reflects the effects of hyperglycemia on the endothelium and the fact that there may be a platelet-vWF interaction, which would explain the atherosclerosis acceleration in these patients.

Taking into account the fact that von Willebrand Factor represents a marker of the endothelial injury and that the functioning of the system of the protein C depends also on the morphological and functional integrity of the vascular endothelium, the indirect correlation that we obtained between these two parameters (*r* = −0.70) is by no means surprising.

Even unexposed to injury factors, the morphofunctionally intact endothelium synthesizes and releases the von Willebrand factor and PAI-1, but, nevertheless, the antithrombotic mechanisms prevail at the endothelial level. However, when endothelium is injured, as, for instance, under the action of certain endotoxins, by means superoxide radicals released from leucocytes, or when they are stimulated by proinflammatory cytokines (IL-1, TNF), there is an accelerated release of thromboplastin and of von Willebrand Factor. Moreover, the endothelium reduces the fibrinolytic activity by the marked increase in the synthesis and release of PAI-1.

The indirect correlation between PC and PAI-1 (*r* = −0.30) evidenced by our study shows an increased risk for thrombotic events to this category of patients.

As for the group of diabetic patients without ischemic cardiopathy (DM − IC), one may assume the existence of a vascular involvement with subclinical symptoms, which is also supported by the fact that endotheliopathy undoubtedly precedes not only the occurrence of cardiovascular conditions but also the very onset of diabetes mellitus. This is due to the fact that the synthesis and release of glycoproteins originating in the endothelium are stimulated by metabolic anomalies [13].

### 3.3. Groups Non DM + IC

In these groups of patients, we have recorded the most significant alterations of the HDLc and LDLc values. They are an important cardiovascular risk factor in both sexes. 

The following most important correlations are noticed between the analyzed parameters.

- Females:
  - indirect correlations between:
    - PAI-1 and HDLc (*r* = −0.52).
  - direct correlations between:
    - vWF and PAI-1 (*r* = 0.88).
- Males:
  - no indirect correlations have been registered;
  - direct correlations between:
    - vWF and PAI-1 (*r* = 0.71).

The results obtained in this group of patients highlight a direct correlation between vWF and PAI-1, a similar aspect met in the groups of diabetic patients.

It has been proven that dyslipidemia is an independent cardiovascular risk factor, and only one in four patients requiring hypolipidemic therapy reaches the recommended target values, in today's worldwide medical practice [17]. Consequently, it is by screening, early diagnosis and intensive dyslipidemia therapy in DM patients that the risk of coronary events may be reduced, that atherosclerotic injury progress may be diminished or prevented, and that already existing atherosclerotic injuries may possibly be recovered.

In many types of vascular-endothelial injuries, a drop in the fibrinolytic capacity of the endothelium was recorded, either by plasminogen activator synthesis decrease or by an increase in the amount of PAI-1 released after the injury, an idea confirmed in our study by the direct correlation between vWF and PAI-1, both in females and males.

Small and dense LDLc particles seem to be the marker of a series of anomalies including HDLc concentration decrease, apoB concentration increase, insulin sensitivity decrease, and procoagulant changes (PAI-1 increase). 

The observations on the atherogenic lipoprotein phenotype and its association with different other proatherogenic alterations have raised the hypothesis of specific genetic interdependences between them. The oxidized LDLc has the following effects: it inhibits the NO and the prostacyclin and disturbs the basic vascular tonus; it stimulates the endothelial cells to release a number of active biological factors (the chemotactic protein for monocytes (MCP-1), the molecules of endothelium-leucocytes adhesion (ELAM), and growth factors; it modifies the aggregability of thrombocytes; it may activate the T leukocytes in the atherosclerosis lesion; it may stimulate the proliferation of smooth muscular cells by inducing the expression and the genetic codification of the growth factor derived from the thrombocyte (PDGF) [18]. Arguments for considering the LDLc oxidized as an essential factor in producing the early atheromatous lesions are the presence in high quantity of the oxidized LDLc at the atherosclerotic lesion site and the capacity of the vascular wall cells (monocytes, macrophagous, endothelial cells, and smooth vascular muscular cells) to produce reactive oxygen species (O_2_^•−^, H_2_O_2_) and to induce LDLc oxidation. 

Researchers proved, in 1990, that the genetic locus responsible for the occurrence of small and dense LDLc is associated with high levels of TG, apoB, with VLDL and IDL masses, and with HDLc and apoA1 decrease. Moreover, genetic linkage studies showed that the LDLc particle sizes as well as the HDLc and LDLc values present positive correlations with an allele of the hepatic lipase gene. The genetic mutations occurring in the locus of the CTEP (cholesteryl ester transfer protein) on chromosome 16 may influence the LDLc sizes and TG values, due to the role it plays in the reverse cholesterol transport. Peroxisome proliferator-activated receptors (PPAR*α*) play an important role in lipid metabolism. In the liver, PPAR*α* activation regulates the gene expression of apoAI, AII, and CIII and of specific endoenzymes involved in *β*-oxidation of fatty acids, whereas PPAR*α* activation in the macrophages induces, through complex mechanisms, the expression of the receptors that HDLc interact with during the reverse cholesterol transport process. Recent data supports PPAR*α* involvement in inflammatory response modulation in the arterial endothelium [19].

However, in nondiabetic patients with ischemic cardiopathy (IC), as the endothelial dysfunction is less severe or even absent, the evolution of these parameters was positive after the delivery of the anticoagulant therapy.

To conclude, dyslipidemia [20] is a frequent condition of diabetes and may contribute to the increase of the cardiovascular risk in these patients.

## 4. Conclusions

The results of our study concerning the researches on lipid metabolism, the proinflammatory activity of plasma, and coagulation parameters have shown that type 2 diabetes mellitus registers a significant frequency in cumulating the main factors of vascular and atherosclerotic risk, the umoral dyslipidemic syndrome (which is characterized by the LDLc increase and HDLc decrease), and the increase in inflammatory markers (IL-1 beta), age, and duration of diabetes, encouraging the increased risk for thrombotic events with lethal potential (increase of vWF, PAI-1, decrease of PC).

Three mechanisms may link the inflammation to the emergence of the diabetes mellitus:

1. the discovery in diabetic patients of the expression of a new Tanis gene, the protein product of which links seric amiloid A;
2. the correlation between the inflammatory process and the level of insulinemia according to a trial on rodents, where the administration of the salicylate led to obesity prevention and insulin resistance [21];
3. the most interesting mechanism, based on the observation that the adiponectin (a protein specific to the adipose tissue which has strong anti-inflammatory properties) [16] is found in small concentrations in obese persons, its concentration increasing as body weight decreases.

Studies in the literature have shown that increased level of inflammatory markers is an independent predictive factor of cardiovascular disease and also that their high plasma levels are correlated with the risk of developing of these clinically manifest diseases. The significantly increased values of interleukin-1-beta evidenced by our study in the groups of diabetic patients require the inclusion of its determination, as well as of other inflammatory markers, in the battery of tests aimed at the prophylaxis of thrombotic events. Beside the inappropriate glycemic control, the insulinresistance, the consecutive hyperinsulinism, an increased flow of fat acids, and proinflammatory cytokines released from the visceral adipose tissue, with direct access to the liver, could induce the procoagulant modifications identified in this study. Hemostasis abnormalities revealed by our study include the increased values of von Willebrand Factor and PAI-1 and decreased levels of protein C. Taking into account the fact that a significant modification of the plasmatic levels of protein C, von Willebrand Factor and PAI-1 could be identified at diabetic patients without clinically manifest cardiovascular diseases, these modifications with prothrombotic potential have been considered to precede the thrombotic complications, having a predictive role for such complications. Considering the significant reduction in the plasmatic levels of the natural coagulation inhibitors at diabetic patients, the synthesis of these protease inhibitors is construed to be affected by the metabolic abnormalities that characterize diabetes, while the enzymatic glycosylation could reduce their anticoagulant effect. In practice, although the assessment of the haemostatic factors does not stand for a routine conduct yet, it would be important for the clinician to know what factors should be explored and to what extent the correction or treatment of the other associated diseases can improve the haemostasis disorders. The procoagulant modifications signaled in this study sum with the deficit of the fibrinolytic activity and with the hyperactivity of platelets, while the risk of diabetic patients' thrombotic complications must also be evaluated in this context too. Concerning the atherothrombotic process, there is a constant connection between the atherogenic lipoproteins (especially oxidized LDLc and LP(a)) and the vascular endothelium. This relation focuses especially on the depressed fibrinolysis phenomenon, hence, the balance deviation between coagulation and fibrinolysis in favor of the former, and the occurrence of thrombotic accidents. The large number of patients with type 2 diabetes mellitus and macrovascular disease requires the investigation of carbohydrate metabolism in nondiabetic patients with macrovascular disease in different locations, even if they had initially normal blood sugar levels, as macrovascular disease may occur even before the alterations of glucose values. All these data support the hypothesis that nondiabetic glucose levels are a continuous risk factor for cardiovascular disease.

 Based on the above mentioned arguments, it may be suggested that the abnormalities of the glycemic balance “per se” may generate prothrombotic modifications of the haemostatic balance. Type 2 diabetes mellitus and its chronic complications, especially the macrovascular disease, take an important place in the medical care system, reducing the life quality of patients suffering from this medical disorder. Only the reduced glycemia values are not enough in order to reduce the rate of complications. The targets of the preventive therapeutic interventions must refer to all the components of the risk factors, including the measures needed to increase the quality of a healthy life. Precocious prevention of the diabetic macrovascular disease must be individualized for each patient, and it must be achieved in parallel to the precocious diagnosis of diabetes mellitus.

## Figures and Tables

**Table 1:** The repartition of the patients by age and gender.

Age	Number of patients between 61–70 years old	Number of patients between 71–82 years old
Female	49	49
Male	36	46

**Table 2:** The groups of patients taken in the study.

	Diabetic patients with clinical or off-clinical disorders related to ischemic cardiopathy (number of patients) F/M	Diabetic patients without clinical or off-clinical disorders related to ischemic cardiopathy (number of patients) F/M	Nondiabetic patients with ischemic cardiopathy-related disorders (number of patients) F/M
Total number of patients (F/M)	**70** 41/29	**70** 37/33	**40** 20/20

Age between 61 and 70 years (F/M)	**42** 24/18	**28** 15/13	**15** 10/5

Age between 71 and 82 years (F/M)	**28** 17/11	**42** 22/20	**25** 10/15

F: female, M: male.

**Table 3:** The mean values of monitored parameters in the studied groups.

Group	Gender	vWF (%)	IL-1*β* (ng/mL)	HDLc (mg/dL)	LDLc (mg/dL)	C Prot (%)	PAI-1 (kU/L)
		$\overset{-}{x}$ ± SD	$\overset{-}{x}$ ± SD	$\overset{-}{x}$ ± SD	$\overset{-}{x}$ ± SD	$\overset{-}{x}$ ± SD	$\overset{-}{x}$ ± SD
DM + IC	F versus M	232.1 ± 30.87	16.11 ± 7.51	55.11 ± 7.99	137.2 ± 24.32	56.3 ± 17.09	15.06 ± 3.81
	*P* > 0.05	224.8 ± 64.00	14.37 ± 6.38	56.44 ± 8.94	144.7 ± 37.06	46.8 ± 7.90	15.2 ± 2.74
DM − IC	F versus M	202.0 ± 52.95	14.47 ± 6.74	57.41 ± 12.41	142.1 ± 36.81	65.4 ± 15.02	13.3 ± 3.59
	*P* > 0.05	184.4 ± 59.56	13.84 ± 7.06	58.78 ± 7.87	140.6 ± 37.14	60.1 ± 3.07	12.3 ± 3.13
Non DM + IC	F versus M	185.7 ± 67.79	5.68 ± 2.78	53.45 ± 13.36	169.9 ± 11.94	74.0 ± 25.27	10.8 ± 4.26
	*P* > 0.05	165.6 ± 75.09	6.74 ± 3.91	52.46 ± 7.22	170.4 ± 24.49	73.1 ± 17.02	10.0 ± 2.62

DM + IC: patients with diabetes mellitus and ischemic cardiopathy.

DM – IC: patients with diabetes mellitus without ischemic cardiopathy.

Non DM + IC: patients without diabetes mellitus and with ischemic cardiopathy.

F: female, M: male.

**Table 4:** The correlation matrix between the monitored parameters in patients from DM + IC group (according to the coefficient of Pearson correlation-*r*).

Parameter	vWF (%)	IL-1*β* (ng/mL)	HDLc (mg/dL)	LDLc (mg/dL)	C Prot (%)	PAI-1 (kU/L)	
	*r*	*r*	*r*	*r*	*r*	*r*	
vWF (%)	1	0.38	−0.33	0.11	−0.11	−0.38	Males
IL-1*β* (ng/mL)	**−0.54**	1	0.17	**−0.42**	0.37	−0.12	
HDLc (ng/mL)	0.23	**−0.7**	1	−0.11	−0.03	0.27	
LDLc (ng/mL)	0.32	−0.35	0.33	1	**−0.88**	−0.19	
C Protein (%)	**−0.7**	−0.48	**−0.56**	−0.42	1	0.08	
PAI-1 (kU/L)	0.14	**0.66**	**−0.53**	−0.4	−0.12	1	
	Females						

**Table 5:** The correlation matrix between the monitored parameters in patients from DM − IC group (according to the coefficient of Pearson correlation-*r*).

Parameter	vWF (%)	IL-1*β* (ng/mL)	HDLc (mg/dL)	LDLc (mg/dL)	C Prot (%)	PAI-1 (kU/L)	
	*r*	*r*	*r*	*r*	*r*	*r*	
vWF (%)	1	−0.37	−0.12	0.12	0.2	0.16	Males
IL-1*β* (ng/mL)	−0.33	1	**0.55**	0.24	−0.28	0.38	
HDLc (ng/mL)	0.19	0.11	1	−0.38	−0.25	−0.13	
LDLc (ng/mL)	−0.2	−0.39	0.28	1	0.3	0.21	
C Protein (%)	**−0.7**	0.2	0.29	−0.05	1	−0.33	
PAI-1 (kU/L)	−0.45	0.13	−0.23	0.22	0.14	1	
	Females						

**Table 6:** The correlation matrix between the monitored parameters in patients from non DM + IC group (according to the coefficient of Pearson correlation-*r*).

Parameter	vWF (%)	IL-1*β* (ng/mL)	HDLc (mg/dL)	LDLc (mg/dL)	C Prot (%)	PAI-1 (kU/L)	
	*r*	*r*	*r*	*r*	*r*	*r*	
vWF (%)	1	0.22	**0.62**	−0.05	0.13	**0.71**	Males
IL-1*β* (ng/mL)	**0.6**	1	0.38	**0.49**	0.53	0.02	
HDLc (ng/mL)	−0.48	−0.47	1	0.02	0.53	**0.5**	
LDLc (ng/mL)	0.3	0.29	−0.21	1	0.1	−0.06	
C Protein (%)	−0.4	−0.4	−0.15	0.42	1	0.2	
PAI-1 (kU/L)	**0.88**	**0.7**	**−0.52**	0.47	−0.24	1	
	Females						

## Abbreviations

DM: Diabetes mellitus
DM + IC: Diabetic mellitus with cardiovascular disease
DM − IC: Diabetic mellitus without cardiovascular disease
ELAM: The molecules of endothelium-leucocytes adhesion
HDLc: High-density lipoprotein cholesterol
IC: Ischemic cardiopathy
IL-1*β*: Interleukin-1-beta
LDLc: Low-density lipoprotein cholesterol
LP: Lipoproteins
MCP-1: The chemotactic protein for monocytes-1
NF-jB: Transcription factor-jB
NO: Nitric oxide
PAI-1: Plasminogen activator inhibitor type 1
PDGF: The growth factor derived from the thrombocyte
PKC: Protein kinase C
PPAR*α*: Peroxisome proliferator-activated receptors
RAGE: The receptor for advanced glycation end products
SD: Standard deviation
TG: Triglyceride
VLDL: Very low-density lipoprotein
vWF: von Willebrand Factor.
